# Wing extension–flexion coupled aeroelastic effects improve avian gliding performance

**DOI:** 10.1098/rsif.2024.0753

**Published:** 2025-05-07

**Authors:** Jasmin C. M. Wong, Vaibhav Joshi, Rajeev K. Jaiman, Douglas L. Altshuler

**Affiliations:** ^1^ School of Civil, Aerospace, and Design Engineering, University of Bristol, Bristol BS8 1TR, UK; ^2^ Department of Mechanical Engineering, Birla Institute of Technology & Science Pilani, K K Birla Goa Campus, Zuarinagar, Goa 403726, India; ^3^ Department of Mechanical Engineering, University of British Columbia, Vancouver V6T 1Z4, Canada; ^4^ Department of Zoology, University of British Columbia, Vancouver V6T 1Z4, Canada

**Keywords:** wing morphing, feathers, fluid–structure interaction, aerodynamics, birds

## Abstract

During flight, birds instigate remarkably large changes in wing shape, commonly termed ‘wing morphing’. These changes in shape, particularly extension–flexion, have been well documented to influence the production of aerodynamic forces. However, it is unknown how wing stiffness changes as a result of the structural rearrangements needed for morphing. We address this gap in knowledge through mechanical testing of *in situ* flight feathers in anaesthetized pigeons and found that while the most distal portion of the feathered wing remained unaffected, proximal areas saw an increase in out-of-plane stiffness due to wing folding. Following this, we used computational fluid–structure interaction simulations to evaluate how this morphing-coupled change in stiffness might modulate local flow patterns to affect aerodynamic performance. We found that flexible wings perform better than entirely rigid wings as an increase in near-wall vorticity delayed flow separation. Furthermore, an increase in stiffness in a folded wing during high-speed flight prevented the reduction in lift seen in more flexible cases caused by aeroelastic flutter modes destructively interfering with shed leading-edge vortices. Collectively, these results reveal that mechanical changes coupled with wing morphing can provide a speed-dependent mechanism to enhance flight performance.

## 1. Introduction

Flight is an efficient way to travel large distances [1] and most avian species employ this form of locomotion and adapt it throughout different tasks and environmental conditions. This adaptability is attributed to wing morphing—the ability to actively change the three-dimensional shape of their wings. By enacting large changes in wing shape, birds are able to fly more efficiently throughout a range of speeds [2–4] and adapt flight dynamics [5–7]. This wing morphing is made possible through multiple structural changes, including to the wing’s soft tissues and feathers, and the contribution of these changes to aerodynamics is not well understood. We address these effects in this study using a mixture of experimental and computational techniques.

Avian wing morphing is actuated by muscles localized in the leading edge of the wing acting on the skeleton [8,9]. As a result of this movement, the flight feathers, flexible passive structures that make up most of the lifting surface of the wing, are rearranged due to their attachment at the base to the skeletal elements [10]. Both the soft tissue anchoring the base of the flight feathers and the interaction between neighbouring feathers have been found to play a role in maintaining the wing structure throughout morphing against aerodynamic loads [11,12].

While the mechanical properties of individual feathers have been relatively well studied [11,13–15], little is known about the effect of the soft tissue and feather–feather interaction on the mechanical properties of the whole wing, particularly throughout morphing. The feathered wing experiences large deformations during flight [16], and morphing-induced changes to the mechanical properties can alter the behaviour of this fluid–structure response. This is particularly significant at the intermediate flow regimes of bird flight where even small changes to the airflow around the wing can have large effects on the aerodynamics [17]. Therefore, insights into wing morphing-coupled aeroelastic effects on the surrounding flow can help further our understanding of how birds use a flexible, multi-unit structure to control flight.

This study investigates this morphing-coupled flow control in the context of gliding flight at different speeds. Birds extend their wings at low speeds and fold their wings at high speeds [2]. We use a two-step process to investigate how this wing extension–flexion can affect aerodynamic performance during gliding flight at different speeds through aeroelastic mechanisms. In the first step, we quantified how wing extension–flexion changed local stiffness in an *in situ* wing and determined which mechanisms drive those changes in stiffness. In the second step, we used computational methods for fluid–structure interactions to estimate how trends observed in the first step would affect the flow behaviour and, subsequently, the aerodynamic performance.

## 2. Methods

### 2.1. *In situ* mechanical testing

#### 2.1.1. Animal procedures

Rock pigeons (*Columba livia*) were acquired from a local breeder (Aldergrove, B.C., Canada) (*n* = 7, mass ranged from 344 to 568 g). On the day of the testing, the pigeon was weighed and then anaesthetized using an isoflurane/oxygen mixture delivered at 1 L min^−1^. This was done first with a facemask delivering 4% isoflurane before being replaced, once the bird entered the surgical plane, by an oral/nasal intubation tube (outer ⊘: 3.3 mm, inner ⊘: 2.5 mm, length: 98 mm) lubricated with Muko Lubricating Jelly (Cardinal Health Canada Ink, Canada). Throughout the procedure, the anaesthesia was reduced to 0.8–1.5% isoflurane to maintain the surgical plane. The pigeon’s awareness and well-being were continuously assessed using a heart rate monitor (Model 2500A VET, Nonin Medical Inc., USA) attached to the foot, an EMMA capnograph (Masimo Corporation, USA) integrated into the intubation tube path and a rectal thermometer for body temperature.

#### 2.1.2. Out-of-plane bending tests

**Figure 1.**
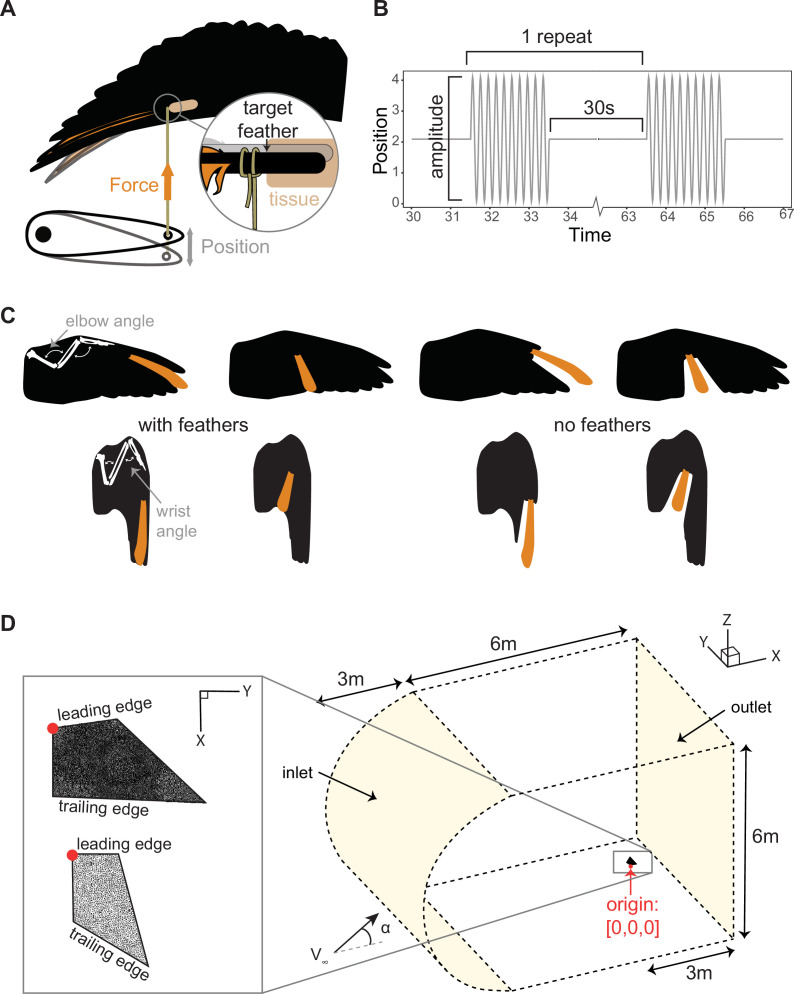
Experimental methods to quantify the mechanical properties of a pigeon wing and models for computational fluid–structure interaction simulations. (A) An anaesthetized pigeon was placed ventral side up and the base of a feather was tethered to a servomotor arm. The arm actuated a position change (grey arrow) which displaced the attached target feather while recording the tension force (orange arrow) generated along the string as the feather resisted this displacement. (B) A sample of the 5 Hz position change actuated by the servomotor. (C) The pigeon wing was positioned in an extended (upper) or folded (lower) position with and without neighbouring feathers. The elbow angle was defined as the acute angle formed by the humerus and the radius bones and wrist angle as the acute angle formed by the ulna and carpometacarpus bones. A distal and a proximal feather were tested (highlighted in orange). (D) A simplified extended and folded wing (insert) was modelled and meshed with 2.5 (upper example mesh) to 5 mm (lower example mesh) within a larger fluid volume for simulation.

The pigeon was placed ventral side up on a stage with its left wing held in place for the bending tests. The target feather was fixed using a non-compliant beading string to the arm of a dual-mode lever system (Model 305C-LR, Aurora Scientific Inc., Canada) (figure 1A). A protocol written and deployed by Dynamic Muscle Control (Version 5.3, Aurora Scientific, Canada) prescribed a 4 mm peak-to-peak amplitude sinusoidal displacement at 5 Hz for 10 cycles via the arm. This signal was repeated seven times with a 30 s pause between each repeat (figure 1B). Simultaneously, the system would record at 100 Hz the force exerted on the lever arm through the string as the feathers’ mechanics resisted this movement.

To test the effect of wing morphing on out-of-plane bending stiffness, the left wing was held in an extended (elbow angle $=90^{\circ }$, wrist angle $=135^{\circ }$) or folded position (elbow angle $=50^{\circ }$, wrist angle $=60^{\circ }$) (figure 1C) non-invasively using a non-compliant beading string to pull the leading primary feather cranially while two vertical steel rods provided counter pressure caudally along the leading edge at the wrist and carpometacarpus. Wing rotation was restricted by stoppers fixed to the two steel rods above and below the leading edge. We made measurements in either the distal or proximal part of the wing by testing the ninth primary, P9, and the first primary, P1, respectively. Furthermore, to differentiate between soft tissue and feather interaction effects on force–displacement responses, the wing was first left intact (both effects are present) and then had all neighbouring feathers clipped at the base (no feather interaction effects) (figure 1C).

### 2.2. Analysis of the wing’s mechanical properties

The raw force–displacement data output by the Dynamic Muscle Control software were pre-processed for analysis. A ‘loop’ was defined as one repeat consisting of 10 cycles, and within each loop, data were classified as ‘loading’ and ‘unloading’. All analysis was done in R [18].

The unnormalized flexural stiffness (k) was calculated to give an intuitive understanding of rigidity, or how much resistance the feathered wing offers against bending. Due to experimental variation in the pre-load tension on the string as a result of the tightness of the clove hitch around the feather shaft, only the data from −0.5 to 2 mm of the position range were considered to prevent underestimation of stiffness caused by initial slack in the string. A line was fit to the remaining data and stiffness was determined by the slope of this line during both loading $\left(k_{l}\right)$ and unloading $\left(k_{u}\right)$. The nonlinear force–displacement behaviour of biological materials (electronic supplementary material, figure S1) means that this metric is only an approximation of the rigidity after a certain degree of pre-load.

To address the first step of our research goal in which we quantify the effect of wing extension–folding on the wing’s mechanical properties, we evaluated the per cent change (Δk) in both loading $\left(\Delta k_{l}\right)$ and unloading $\left(\Delta k_{u}\right)$ conditions:

(2.1) $$\begin{array}{r} \Delta k_{\text{l}}=\frac{k_{\text{l,folded}}-k_{\text{l,extended}}}{k_{\text{l,extended}}}\times 100\%\\ \Delta k_{\text{u}}=\frac{k_{\text{u,folded}}-k_{\text{u,extended}}}{k_{\text{u,extended}}}\times 100\% \end{array}$$

where $k_{extended}$ and $k_{folded}$ are extended wing stiffness and folded wing stiffness, respectively, in either loading $\left(k_{l}\right)$ or unloading $\left(k_{u}\right)$ conditions.

We then built a linear mixed-effects model using the *lmerTest* package [19] to evaluate the significance of the changes in wing mechanical properties. We tested the following five models explaining k and four models explaining Δk:

(i) k or Δk is a function of the fixed effects of feather–feather interaction, feather location, wing position (not available for Δk) and the uncontrolled random effects of individual variation;
(ii) k or Δk is a function of the fixed effect of feather–feather interaction and the uncontrolled random effects of individual variation;
(iii) k is a function of the fixed effect of wing position and the uncontrolled random effects of individual variation;
(iv) k or Δk is a function of the fixed effect of feather location and the uncontrolled random effects of individual variation;
(v) k or Δk is only a function of the uncontrolled random effects of individual variation.

Akaike information criterion (AIC) weights were used to find the best model (electronic supplementary material, tables S1, S3, S5, S7). Then, *p*-values were used to determine whether the effect of each of the treatments was significantly different from the null hypothesis (electronic supplementary material, tables S2, S4, S6, S8). Confidence intervals, calculated using the *lme4* package [20], were used to determine the significance of the difference in effects between treatments, and $R^{2}$ values were used to determine how much of the variation is explained by the independent variables contained in the best fit model.

### 2.3. Computational fluid–structure modelling

#### 2.3.1. Building the model

We used computational simulation to evaluate how increasing or decreasing wing stiffness during wing extension–flexion can affect the near-field flow behaviour through aeroelastic response. To reduce the challenges involved in the fluid–structure interaction simulation of a complex, anisotropic, multi-material structure, we modelled the wing as an isotropic, polygonal flat plate with a constant thickness (figure 1D).

To get a sense of the three-dimensional wing shape of a pigeon, we performed motion tracking on wing specimens. Two pigeon wings were disarticulated at the shoulder from cadavers. Eleven markers were placed on the dorsal side of the wing: four at the humerus head, elbow joint, wrist joint and carpometacarpus to track joint angles, and seven were placed on the leading and trailing edge of the wing (three on the leading edge of the humerus head, elbow joint and carpometacarpus bone, and four on the tips of the S10, S1, P7 and P10 flight feathers) to track wing shape. The OptiTrack motion capture system (Natural Point Inc., USA) was used to record the three-dimensional coordinates of these markers as the wing was moved through its extension–flexion range of motion. For more details on the motion capture methods, see Harvey *et al*. [7].

The elbow angle was calculated based on the position of the markers at the humerus head, elbow joint and wrist joint, and the wrist angle was calculated based on the position of the markers at the elbow joint, wrist joint and carpometacarpus bone. To match the wing positions used during the *in situ* mechanical testing, we selected data closely matching the extended wing joint angles (elbow angle $=90\pm 3^{\circ }$, wrist angle $=135\pm 3^{\circ }$) and the folded wing joint angles (elbow angle $=47\pm 3^{\circ }$, wrist angle $=78\pm 3^{\circ }$).

We selected four key points: the humerus, the leading edge of the carpometacarpus, the tip of P10 and the tip of S10 to best approximate the wing shape as a four-sided polygon. The selected data were then used to generate coordinates for a two-dimensional outline of the wing planform. Four key points were extracted: the humerus $\left\{x_{hum},y_{hum},z_{hum}\right\}$, the leading edge of the carpometacarpus $\left\{x_{cmc},y_{cmc},z_{cmc}\right\}$, the tip of P10 $\left\{x_{p10},y_{p10},z_{p10}\right\}$ and the tip of S10 $\left\{x_{s10},y_{s10},z_{s10}\right\}$, each with their respective x,y,z, coordinates. We first flattened these four coordinates onto the same plane, which was defined by the humerus, the leading edge of the carpometacarpus and the tip of P10. To do this, we let $\vec{v_{1}}$ be the vector from the leading edge of the carpometacarpus to the humerus:


$$\vec{v_{1}}=\langle x_{hum}-x_{cmc},y_{hum}-y_{cmc},z_{hum}-z_{cmc}\rangle$$

and $\vec{v_{2}}$ be the vector from the leading edge of the carpometacarpus to P10:


$$\vec{v_{2}}=\langle x_{p10}-x_{cmc},y_{p10}-y_{cmc},z_{p10}-z_{cmc}\rangle .$$

The unit normal to this plane was then defined as


$$\hat{n}=\frac{\vec{v_{1}}\times \vec{v_{2}}}{||\vec{v_{1}}\times \vec{v_{2}}||}=\langle n_{i},n_{j},n_{k}\rangle .$$

We then projected the coordinates of the S10 marker onto this defined plane by letting $\vec{v_{3}}$ be the vector from the leading edge of the carpometacarpus to S10:


$$\vec{v_{3}}=\langle x_{s10}-x_{cmc},y_{s10}-y_{cmc},z_{s10}-z_{cmc}\rangle$$

and calculated the projected coordinates of S10 as follows:


(2.2) $$\begin{array}{lrlr} & d & =\vec{v_{3}}\cdot \hat{n} & \end{array}$$

(2.3) $$\begin{array}{r} \{x_{s10,proj},y_{s10,proj},z_{s10,proj}\}=\{x_{s10}-dn_{i},y_{s10}-dn_{j},z_{s10}-dn_{k}\}. \end{array}$$

Next, we rotated the coordinates so that they all lay on the *xy* plane of the global coordinate frame. This was done by finding the axis of rotation:


(2.4) $${\vec{n}}_{rot}=\hat{n}\times \hat{k}=\langle n_{rot,i},n_{rot,j},n_{rot,k}\rangle$$

where $\hat{k}=\left\langle 0,0,1\right\rangle$. The sine of the angle of rotation was defined as $s=||\vec{n_{rot}}||$ and the cosine of the angle of rotation was defined as $c=\hat{n}\cdot \hat{k}$. Then, the rotation matrix (R) was calculated as


(2.5) $$\text{R}=\text{I}+\text{SS}+{\text{SS}}^{2}\frac{1}{1+c},$$

where I is the identity matrix defined as $\text{I}=\left(\begin{array}{ccc} 1 & 0 & 0\\ 0 & 1 & 0\\ 0 & 0 & 1 \end{array}\right)$, and SS is the skew symmetric cross-product of $\vec{n_{rot}}$ defined as $\text{SS}=\left(\begin{array}{ccc} 0 & -n_{rot,k} & n_{rot,j}\\ n_{rot,k} & 0 & -n_{rot,i}\\ -n_{rot,j} & n_{rot,i} & 0 \end{array}\right)$. The rotated coordinates for each point were then calculated by multiplying the original coordinates with the rotation matrix:


(2.6) $$\{x_{rotxy},y_{rotxy},z_{rotxy}\}=\text{R}\left\{\begin{array}{c} x_{proj}\\ y_{proj}\\ z_{proj} \end{array}\right\}$$

where $\left\{x_{proj},y_{proj},z_{proj}\right\}$ are the coordinates of the respective four markers after the projection of the S10 point. We also rotated the coordinates around the *z*-axis so that the root of the wing was parallel to the *x*-axis. The new coordinates were then calculated by


(2.7) $$\{x_{rotxyz},y_{rotxyz},z_{rotxyz}\}=R_{y}\left\{\begin{array}{c} x_{rotxy}\\ y_{rotxy}\\ z_{rotxy} \end{array}\right\},$$

where $R_{y}=\left[\begin{array}{cc} \text{cos}\theta & -\text{sin}\theta \\ \text{sin}\theta & -\text{cos}\theta \end{array}\right]$, and θ is the angle between the root vector and the global *x*-axis.

Finally, we translated all the coordinates so that the humerus position lay at the origin {0,0,0} to ensure that all wings were positioned at the same location within the fluid volume. For the folded wing, there was an additional transformation where we scaled the coordinates so that the root chord $\left(c_{r}\right)$ matched that of the extended wing by multiplying the original coordinates by the scaling factor:


(2.8) $$s=\frac{c_{r,e}-c_{r,f}}{c_{r,f}+1},$$

where $c_{r,f}$ is the original root chord of the folded wing and $c_{r,e}$ is the root chord of the extended wing.

#### 2.3.2. Prescribing the structural parameters

Once the coordinates defining the planform area $\left\{x_{rotxyz},y_{rotxyz},z_{rotxyz}\right\}$ were defined, the thickness was defined to generate a three-dimensional wing. This was done by importing these coordinates into Gmsh (v. 2.13.0 for Linux), a three-dimensional finite element mesh generator [21]. The coordinates were duplicated and projected upwards by the wing thickness (h), which was adjusted to ensure solver success for different flow conditions. The model’s overall mechanical rigidity was a function of h and the prescribed Young’s modulus $\left(E^{{,}^{\prime }}\right)$. Since it was not possible to build a model with a thickness (h) matching the average thickness of a real pigeon wing $h_{real}=0.0036m$ , $E^{{,}^{\prime }}$ was adjusted to ensure aeroelastic similarity to a real pigeon wing with a thickness $h_{real}$ and a Young’s modulus (E). The aeroelastic similarity was defined using the aeroelastic number Æ:


(2.9) $$\text{Æ}=\frac{Eh_{real}}{\frac{1}{2}\rho_{f}V_{\infty }^{2}c_{mac}}.$$

If we set


(2.10) $${\text{Æ}}_{real}={\text{Æ}}_{model}$$

using the definition in (2.9) while density $\left(\rho_{f}\right)$, free-flow velocity $\left(V_{\infty }\right)$ and mean aerodynamic chord $\left(c_{mac}\right)$ remain the same between real and model conditions, then (2.10) becomes


(2.11) $$Eh_{real}=E^{\prime }h$$

and $E^{{,}^{\prime }}$ can be prescribed to the wing model with a thickness h. We chose two E values for our flexible wing models: $E=10^{6}$ (F1) as an intermediary value derived from our experimental testing that accounts for feathers being embedded in compliant soft tissue *in situ*, and $E=10^{9}$ (F2) to reflect the values from previous work on individual, rigidly fixed pigeon feathers [14]. For the F1 estimation, we used the Euler–Bernoulli beam bending equation:


$$E=\frac{kl^{3}}{3I},$$

where I is the second moment of area of a filled circle, l is the length of the feather from the base to the string’s attachment point and k is the stiffness values from the bending tests. Using a range of mean k values, E ranged from an order of magnitude of $10^{5}$ to $10^{7}$. The mean aerodynamic chord $\left(c_{mac}\right)$ was calculated using a custom function in R based on an analytic method outlined by Diehl [22] that iteratively divided the wing planform into increasing number of panels up to a maximum of 200 panels until $c_{mac}$ did not deviate by more than $5\times 10^{-5}$ units.

A model of this wing with the prescribed geometric parameters as detailed in table 1 was placed within a fluid volume made up of a 6 × 6 × 6 m cube combined with a half cylindrical volume upstream to smooth out irregularities in the flow field at the inlet corners (figure 1D). We discretized the fluid volume and the solid volume making up the wing using an unstructured tetrahedral mesh that adapted better to large deformations. We benchmarked our model against three rectangular plate geometries of varying aspect ratios at angles of attack (α) of $0^{\circ }$, $30^{\circ }$, $60^{\circ }$¸$90^{\circ }$ and a Reynolds number of 150 000 as described by Shademan & Naghib-Lahouti [23] (electronic supplementary material, figure S2). This allowed us to ensure that our solver can replicate flow patterns of other studies on rigid flat plates of similar scale and flow speeds as our pigeon model.

**Table 1.** Geometric parameters of Gmsh models.

parameter	extended wing	folded wing
span (b)	0.3732 m	0.1874 m
mean aerodynamic chord $\left(c_{mac}\right)$	0.1597 m	0.1883 m
root chord $\left(c_{r}\right)$	0.1670 m	0.1670 m
distal chord $\left(c_{d}\right)$	0.3003 m	0.2866 m
area (S)	0.0495 m${,}^{2}$	0.0311 m${,}^{2}$
aspect ratio (AR)	11.2522	4.5128
model thickness (h)	0.007–0.02m	0.007–0.02m

#### 2.3.3. Fluid model and boundary conditions

Fluid–structure behaviour was simulated using in-house code to solve the governing incompressible Navier–Stokes equations with a delayed detached eddy simulation (DDES) turbulence model coupled with nonlinear structural equations [24,25]. The DDES model is based on the detached eddy simulation (DES) approach, which combines Reynolds-averaged Navier–Stokes (RANS) for boundary layer regions with large-eddy simulation (LES) for detached eddies. This hybrid approach optimizes computational resources by focusing LES computations on areas with significant turbulent eddies, where the turbulence length scale is greater than the grid dimensions. This makes it suitable for high-Reynolds number flows and complex geometries [26]. The DDES model builds on this by implementing three aspects: (i) modifying the turbulence length scale to delay the onset of LES-like behaviour in attached boundary layers, ensuring RANS is used more robustly in these regions, (ii) using a blending function to transition smoothly between RANS in near-wall regions and LES in separated flow regions, minimizing model inconsistencies, and (iii) using this near-wall treatment to avoid excessive grid refinement while still capturing large-scale flow structures far from the wall [27]. Further explanation and validation of our implementation of the DDES solver can be found in Joshi & Jaiman [28]. These model equations were then discretized using a stabilized Petrov–Galerkin finite element method in an arbitrary Lagrangian–Eulerian (ALE) reference frame [25] as ALE-based finite element methods can accurately handle fluid–structure interaction cases with large deformations and complex kinematics such as those found in nature [29]. A radial basis function was used to interpolate fluid forces and structural displacements along the fluid–solid interface [25], which was simulated using a second-order accurate combined field with explicit interface (CFEI) formulation [30]. The details on the numerical methods and solver can be found in Gurugubelli & Jaiman [24] and Li *et al*. [25], and validation of the solver with cases involving flexible wings for the animal flight can be found in Li *et al*. [31] and Joshi *et al*. [32].

The wing and the fluid volumes were discretized using a tetrahedral mesh with element size ranging from 2.5 to 5 mm at the wing, adjusted to ensure computational convergence given the amount of deformation within each iteration, and increasing towards the fluid boundaries. Overall, the fluid volume contained approximately 1.1 million nodes. Flow patterns around the deformable wing at an $\alpha =-10^{\circ },0^{\circ },10^{\circ },20^{\circ },30^{\circ }$ were solved for cases with the fluid and structural properties listed in table 2. The inlet and the lower surface of the fluid volume were prescribed a free-flow velocity of $V_{\infty }$:


$$V_{\infty }=\langle u,v,w\rangle ,$$

**Table 2.** Fluid and structural properties.

property	extended wing	folded wing
fluid density $\left(\rho_{f}\right)$	1.225 $kg\;m^{-3}$	1.225 $kg\;m^{-3}$
solid density $\left(\rho_{s}\right)$	1060 $kg/m^{3}$	1060 $kg\;m^{-3}$
fluid viscosity (μ)	1.83 Pa.s	1.83 Pa.s
free-flow velocity $\left(V_{\infty }\right)$	10 $m\;s^{-1}$, 20 $m\;s^{-1}$	10 $m\;s^{-1}$, 20 $m\;s^{-1}$
Reynolds number (Re)	107942, 215884	127224, 254448
Poisson ratio (ν)	0.3	0.3
Young's modulus (E)	$10^{6}$ (F1), $10^{9}$ (F2), ∞ (R)	$10^{6}$ (F1), $10^{9}$ (F2), ∞ (R)

where $u=V_{\infty }\text{cos}(\alpha \pi /180)$, v=0, $w=V_{\infty }\text{sin}(\alpha \pi /180)$.

Meanwhile, the outlet and top surfaces of the fluid volume had a pressure outlet boundary condition of zero. The sides of the fluid volume were prescribed planar symmetric slip conditions. All fluid volume surfaces also had ALE boundary conditions of zero meaning that these surfaces did not move due to fluid forces. The wing root also had Dirichlet boundary conditions of u=0,v=0,w=0 and ALE boundary conditions of zero since the wing root in an actual bird would not interact with the fluid. The rigid cases were solved iteratively over time with a time step Δt=0.01s for 10 000 iterations. Due to the additional complexity of a deforming solid, the flexible cases were solved with a time step Δt=0.0001s for 50 000 iterations with the mesh displacement data saved every 50 iterations (sample frequency = 200 Hz). Output files for Tecplot post-processing were created every 1000 iterations. Cases were run on the University of British Columbia’s Advance Research Computing (ARC) Sockeye platform. Each case used 160 processor nodes and 128 GB of memory.

### 2.4. Aerodynamic performance analysis

Using the results from the fluid–structure interaction simulations, we were able to observe the trends in aerodynamic performance caused by increasing or decreasing wing stiffness, wing extension and folding and flight speed. The forces along the global coordinate axes $\left(F_{x},F_{y},F_{z}\right)$ output by the solver were converted into lift (L) and drag (D):


(2.12) $$\begin{array}{l} L=F_{z}\text{cos}\alpha -F_{x}\text{sin}\alpha \end{array}$$

and


(2.13) $$\begin{array}{l} D=F_{x}\text{cos}\alpha +F_{z}\text{sin}\alpha . \end{array}$$

Lift and drag were then normalized as lift $\left(C_{L}\right)$ and drag $\left(C_{D}\right)$ coefficients to allow for comparison between cases of varying stiffness:


(2.14) $$\begin{array}{l} C_{L}=\frac{L}{\frac{1}{2}\rho V_{\infty }^{2}S} \end{array}$$

and


(2.15) $$\begin{array}{l} C_{D}=\frac{D}{\frac{1}{2}\rho V_{\infty }^{2}S}. \end{array}$$

We then quantified performance by evaluating the slope of the linear portion of the $C_{L}∼\alpha$ curve for $-10^{\circ }\leq \alpha \leq 10^{\circ }$, $C_{L\alpha }$ and the maximum lift-to-drag ratio $(C_{L}/C_{D}{)}_{max}$, using data between normalized time $\left(t^{*}\right)$ of 150−250, where


(2.16) $$t^{*}=\frac{tV_{\infty }}{c_{mac}}$$

to ensure that only simulation results from well-established flow were used.

To further explore the mechanisms driving the effects on aerodynamic performance, we looked at both the time-averaged and instantaneous pressure distribution of the fluid–structure responses on the nearby flow. The pressure coefficient


(2.17) $$C_{p}=\frac{\Delta P}{\frac{1}{2}\rho V_{\infty }^{2}S},$$

where ΔP is the difference between the pressure at a given point and the freestream pressure, was visualized throughout a chordwise slice positioned, where the chord $c=c_{mac}$ and values in this slice lying on the wing surface were extracted using Tecplot (version 2013) (Tecplot USA).

We also quantified the aeroelastic deformation through the wing tip’s plunge and twist response using the output displacements. These coordinates were rotated to ensure that $V_{\infty }$ was parallel to the *x*-axis of the global coordinate frame. Plunge response was defined as


(2.18) $$z^{*}=z_{TE}\text{cos}\alpha -x_{TE}\text{sin}\alpha ,$$

where $z_{TE}$ and $x_{TE}$ are the z and x coordinates of the most distal point of the trailing edge. The pitch angle was defined as


(2.19) $$\theta_{p}=\text{arcsin}(\frac{z_{LE}^{*}-z_{TE}^{*}}{c_{d}})$$

in degrees, where $z_{LE}^{*}$ is the rotated z coordinate of the most distal point of the leading edge and $z_{TE}^{*}$ is the rotated z coordinate of the most distal point of the trailing edge. Due to the cyclic nature of these data, we summarized these responses in the frequency domain using the ‘fft’ function from the R *stats* package to perform a discrete Fourier transform using a fast algorithm on the $z^{*}$ or $\theta_{p}$ time-series data [18]. The transformed data, a complex vector H, were normalized by dividing values by the length of the vector, N. The magnitude, |H| was then calculated by taking the modulus of H, and the frequency calculated by f=0,1,...,N×(Fs/N), where Fs is the sampling frequency of $z^{*}$ or $\theta_{p}$. The first two harmonics were identified using the ‘findpeaks’ function in the *pracma* R package [33] on the |H|(f) data.

Vortex behaviour around an aerofoil can play a significant role in aerodynamics as seen in studies on flapping and flutter [34]. We quantified vorticity via the $\lambda_{2}$ criterion that is widely used to indicate vortex cores when negative, and is robust in its identification compared with other algorithms [35]. Despite this, it is important to note that it can fail to fully resolve entire vortex structures as it is dependent on a chosen threshold value [36], particularly in cases with small-scale turbulent fluctuations and high vorticity gradients [37]. Using Tecplot, we plotted the isosurfaces corresponding to $\lambda_{2}=-0.5$, coloured by the normalized velocity $u^{*}=\frac{\sqrt{u^{2}+v^{2}+w^{2}}}{V_{\infty }}$, to visualize this vorticity. $\lambda_{2}$ was calculated using a Tecplot script that first calculated the velocity gradient tensor from the velocity values $\vec{u}(x,y,z,t)=<u,v,w>$ at each node:


(2.20) $$\begin{array}{r} \nabla \vec{u}=\left[\begin{array}{ccc} \partial_{x}u & \partial_{y}u & \partial_{z}u\\ \partial_{x}v & \partial_{y}v & \partial_{z}v\\ \partial_{x}z & \partial_{y}z & \partial_{z}z \end{array}\right]. \end{array}$$

This is then decomposed into a symmetric part, the rate of strain tensor that describes the rate of stretching and shearing


(2.21) $$\begin{array}{rl} S & =\frac{\nabla \vec{u}+\nabla {\vec{u}}^{T}}{2}\\ & =\left[\begin{array}{ccc} \partial_{x}u & \frac{1}{2}(\partial_{y}u+\partial_{x}v) & \frac{1}{2}(\partial_{z}u+\partial_{x}w)\\ \frac{1}{2}(\partial_{y}u+\partial_{x}v) & \partial_{y}v & \frac{1}{2}(\partial_{z}v+\partial_{y}w)\\ \frac{1}{2}(\partial_{z}u+\partial_{x}w) & \frac{1}{2}(\partial_{z}v+\partial_{y}w) & \partial_{z}w \end{array}\right] \end{array}$$

and an antisymmetric part, the vorticity tensor, which describes the rate of rotation:


(2.22) $$\begin{array}{r} \begin{array}{rl} \Omega & =\frac{\nabla \vec{u}-\nabla {\vec{u}}^{T}}{2}\\ & =\left[\begin{array}{ccc} 0 & \frac{1}{2}(\partial_{y}u-\partial_{x}v) & \frac{1}{2}(\partial_{z}u-\partial_{x}w)\\ -\frac{1}{2}(\partial_{y}u-\partial_{x}v) & 0 & \frac{1}{2}(\partial_{z}v-\partial_{y}w)\\ -\frac{1}{2}(\partial_{z}u-\partial_{x}w) & -\frac{1}{2}(\partial_{z}v-\partial_{y}w) & 0 \end{array}\right]. \end{array} \end{array}$$

Finally, we could then use Tecplot’s tensor eigensystem command to find the second eigenvalue $\left(\lambda_{2}\right)$ of $S^{2}+\Omega^{2}$.

## 3. Results

### 3.1. Morphing alters stiffness through feather interactions

The *in situ* mechanical tests show that the structures supporting the tested flight feather responded in a viscoelastic manner with energy-absorbing hysteresis (electronic supplementary material, figure S1) typical of a fibrous biomaterial [38]. The response to a 5 Hz dynamic stimulus was found to be primarily elastic with an average phase lag of 11.56${,}^{\circ }$ between the position and force. There was also negligible variation in the force–displacement response between cycles or repeats but a large variation among individuals (electronic supplementary material, figure S1) due to biological and physiological differences as well as experimental inconsistencies in anaesthetic depth or string tension between the feather and the servomotor arm during testing. Individuals that were more tense could result in an increased ‘holding’ force on the feather [39], and less initial slack in the string could result in stiffness values being calculated from the steeper portion of the nonlinear force-position curve. We accounted for these effects by including each experiment/bird as a random source of variation in statistical analyses.

**Figure 2.**
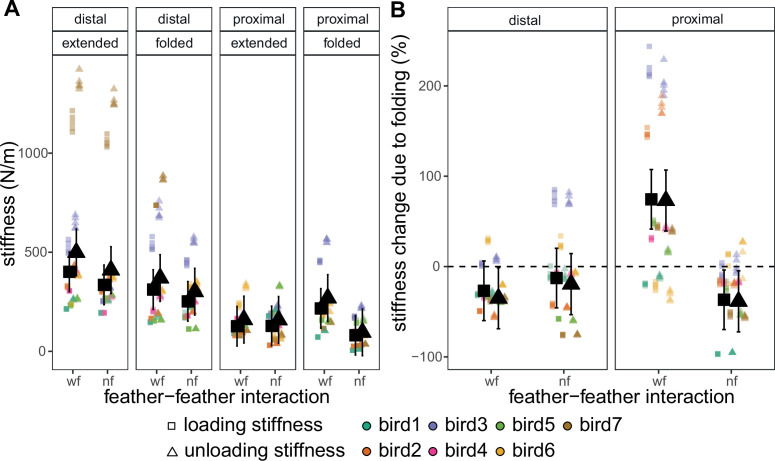
Bending stiffness of the wing varies depending on feather location, wing position, presence of feather interaction (‘wf’) or lack thereof (‘nf’). Individual birds are differentiated by point colour. Group means are represented by solid black points with bars representing 90% confidence intervals. (A) Loading ($k^{\prime }$) (square) and unloading ($k^{§quot;}$) (triangle) modulus. $k^{\prime }:p<0.001,\;R_{marg}^{2}=29.0\%,R_{cond}=57.7\%$; $k^{\prime \prime }:p<0.001,\;$ $R_{marg}^{2}=30.6\%,R_{cond}^{2}=58.8\%$. (B) Per cent change of the $k^{\prime }$ and $k^{\prime \prime }$ due to wing folding. The distal wing experienced a decrease in stiffness (negative change) through folding that was unchanged with the removal of neighbouring feathers. The proximal wing experienced an increase in stiffness (positive change) through folding that was not present with the removal of neighbouring feathers. $\Delta k^{\prime }:p<0.001,\;R_{marg}^{2}=40.7\%,R_{cond}=67.4\%$; $\Delta k^{\prime \prime }:p<0.001,\;R_{marg}^{2}=41.9\%,R_{cond}^{2}=67.4\%$.

While all tests resulted in a similar viscoelastic response, we found that the mechanical bending stiffness during both loading $\left(k_{l}\right)$ and unloading $\left(k_{u}\right)$ varies locally via different mechanisms with wing morphing (figure 2). Statistical analyses revealed that models including feather location, wing position and whether or not feather–feather interaction is present better explain variation in the data compared with models in which variation is attributed only to individual differences $\left(R_{marg}^{2}=29\%,R_{cond}^{2}=57.7\%\text{ for }k_{l};R_{marg}^{2}=30.6\%,R_{cond}^{2}=58.8\%\text{ for }k_{u}\right)$. Overall, wing stiffness was higher in the distal portions of the wing (figure 2A). Due to individual variation, the effects of wing morphing were more apparent when considering the per cent change in local stiffness due to wing morphing (figure 2B). Stiffness only marginally decreased in the distal wing $\left(\Delta k_{l,mean}=-26.83\%,\Delta k_{u,mean}=-35.10\%\right)$ but dramatically increased in the proximal wing $\left(\Delta k_{l,mean}=74.40\%,\Delta k_{u,mean}=73.09\%\right)$ with wing folding. When neighbouring feathers were removed, there was no significant change in the effect in the distal wing $\left(\Delta k_{l,mean}=-12.75\%,\Delta k_{u,mean}=-19.47\%\right)$ but a complete reversal in the effect in the proximal wing $\left(\Delta k_{l,mean}=-36.76\%,\Delta k_{u,mean}=-38.58\%\right)$. As such, wing folding caused a slight decrease in stiffness in the distal wing through an increase in tissue compliance while the proximal wing experienced a large increase in tissue compliance through an increase in feather–feather interaction.

### 3.2. The effect of reduced aspect ratio on aerodynamic performance

Simplified wing models (figure 1D) were used to evaluate the effect of wing folding and the associated structural changes on near-wall flow patterns. Wing folding has been found to improve gliding performance through a change in wing aspect ratio and a subsequent reduction in pressure drag [2]. Before considering the changes in wing stiffness, we considered the effect of geometry, particularly the reduction in aspect ratio caused by wing folding, on the airflow. In agreement with experimental studies, we saw a small reduction in lift and a larger reduction in drag with wing folding (electronic supplementary material, figure S2b). This effect can be observed using rectangular plate models with large differences in aspect ratio such as those found in Shademan & Naghib-Lahouti [23] whose trends were replicated in our study (electronic supplementary material, figure S2). The mean $C_{p}$ chordwise distribution around the wing concurred with previous work that this is indeed due to a reduced pressure drag caused by a smaller low-pressure separation bubble as indicated by the length of the plateau region of the pressure coefficient curves [40] (electronic supplementary material, figure S2c). However, the effect was less apparent in the wing models compared with the rectangular plates (electronic supplementary material, figure S2b) likely due to the effect of wing taper that can reduce this effect [41]. Therefore, we next coupled the effect of wing stiffness to the more obvious geometry changes caused by wing folding to assess if further improvements can be made to aerodynamic performance.

### 3.3. Wing flexibility can improve aerodynamic performance

**Figure 3.**
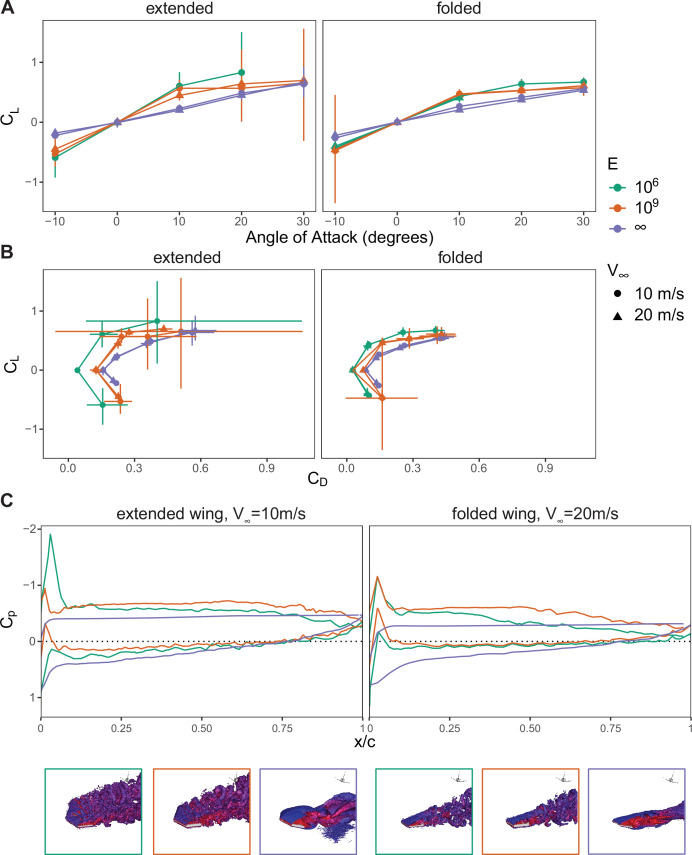
A flexible wing increases lift and decreases drag by increasing vorticity and promoting boundary layer reattachment. Aerodynamic performance metrics are shown by (A) $C_{L}$ as a function of α and (B) $C_{L}$ to $C_{D}$ for an extended and folded wing model. Colours represent different E prescribed to the model and point shape represents the inlet flow speed. Range bars indicate the maximum and minimum values in the data in the steady-state results. (C) Sample chord-wise time-averaged pressure (*C*p) for different E is indicated by the line colour. The corresponding vorticity for each E is shown in an isometric view with isosurfaces corresponding to $\lambda_{2}=-0.5$and coloured by $u^{*}<0.9$ (red) or $u^{*}>1.1$ (blue). In cases with complete flow separation, large turbulent wakes persisted into wider regions around the wing and this $\lambda_{2}$ could only partially resolve some of the vortex structures [36,37].

Both the flexible extended and folded wings demonstrated improved performance compared with the rigid models, with a larger level of improvement in the extended wing (figure 3). At all successfully converged combinations of air speeds and wing shape, the wing with the lowest prescribed *E* (F1) generated the highest lift-to-drag coefficient $(C_{L}/C_{D}{)}_{max}$ (figure 3B, table 3). This increase in lift and decrease in drag were reflected by the higher suction peak in the chordwise pressure distribution (figure 3C and electronic supplementary material, figure S3). At positive angles of attack, the flow separated much closer to the leading edge of the rigid wing resulting in a low-pressure region further back towards the trailing edge of the wing and a large turbulent wake, generating drag (electronic supplementary material, figure S4). On the other hand, the deformation of the flexible wings caused the wing to interact with the separating boundary layer, resulting in small vortex structures and a more attached flow over the dorsal surface of the wing (figures 3C and electronic supplementary material, figure S3, S4). The increase in vorticity and attached flow resulted in a smaller low-pressure area further upstream on the dorsal side of the wing compared with the rigid model (figure 3C). This reoriented the aerodynamic force vectors more in favour of lift and less in favour of drag. It is important to note that in one case, the F1 extended wing exposed to a high air speed failed to converge. This likely occurred due to mesh errors from excessively large deformations over a short time period. While further optimization of the mesh and solver may eventually provide a solution, this result mirrored structural failures experienced by extended fixed wings at similar high speeds in experimental wind tunnel studies [3].

**Table 3.** Lift slope CLα and maximum lift-to-drag (CL/CD)max as a function of wing flexibility (*E*) and wing shape.

wing shape	$V_{\infty }$	E	$C_{L\alpha }$	$(C_{L}/C_{D}{)}_{max}$	$\alpha_{(C_{L}/C_{D}{)}_{max}}$
extended	10 m s^−1^	F1 $\left(10^{6}\right)$	0.0597	4.0375	10
extended	10 m s^−1^	F2 $\left(10^{9}\right)$	0.0548	2.3702	10
extended	10 m s^−1^	R (∞)	0.0226	1.3029	20
extended	20 m s^−1^	F2 $\left(10^{9}\right)$	0.0449	2.3390	20
extended	20 m s^−1^	R (∞)	0.0191	1.2788	20
folded	10 m s^−1^	F1 $\left(10^{6}\right)$	0.0430	4.4276	10
folded	10 m s^−1^	F2 $\left(10^{9}\right)$	0.0473	2.9862	10
folded	10 m s^−1^	R (∞)	0.0263	1.8562	10
folded	20 m s^−1^	F1 $\left(10^{6}\right)$	0.0405	4.4344	10
folded	20 m s^−1^	F2 $\left(10^{9}\right)$	0.0459	2.9316	10
folded	20 m s^−1^	R (∞)	0.0212	1.5793	20

The wing models prioritized replicating the morphing-induced change highlighted by Pennycuick [2], but do not account for the variable camber and thickness found in bird wings, which can affect performance [42]. Our models had a 0% camber compared with an estimated 5% in pigeons [43], likely resulting in lower $C_{L}/C_{D}$ values for all angles of attack [42,44]. As our wing models had the same simplified 0% camber, the trends resulting from varying stiffness in our simulation likely remain relevant for a cambered wing. The wing models also had a constant thickness which ranged from 3.7 to 10.6%, adjusted to ensure computational convergence. These values fall within the range of reported maximum wing thickness of 2.5–10% [43,45]. The F1 wings achieved a higher $C_{L}/C_{D}$ than the F2 wings which could have been caused, in part, by their thinner profile. However, the increase in performance was greater than previously reported values [42,46]. Notably, the R wings performed worse than F1 and F2 wings, despite having equal or greater thickness, indicating that thickness alone cannot explain the trends. It is worth considering that wing thickness effects are sensitive to small changes in Reynolds number [42,46]. Therefore, while our model simplifications are unlikely to alter the qualitative trends described in this study, further work with more realistic and complex flexible models in the Reynolds regime used in this study would be valuable to produce more accurate predictions.

### 3.4. The impact of wing stiffness on vortex dynamics

**Figure 4.**
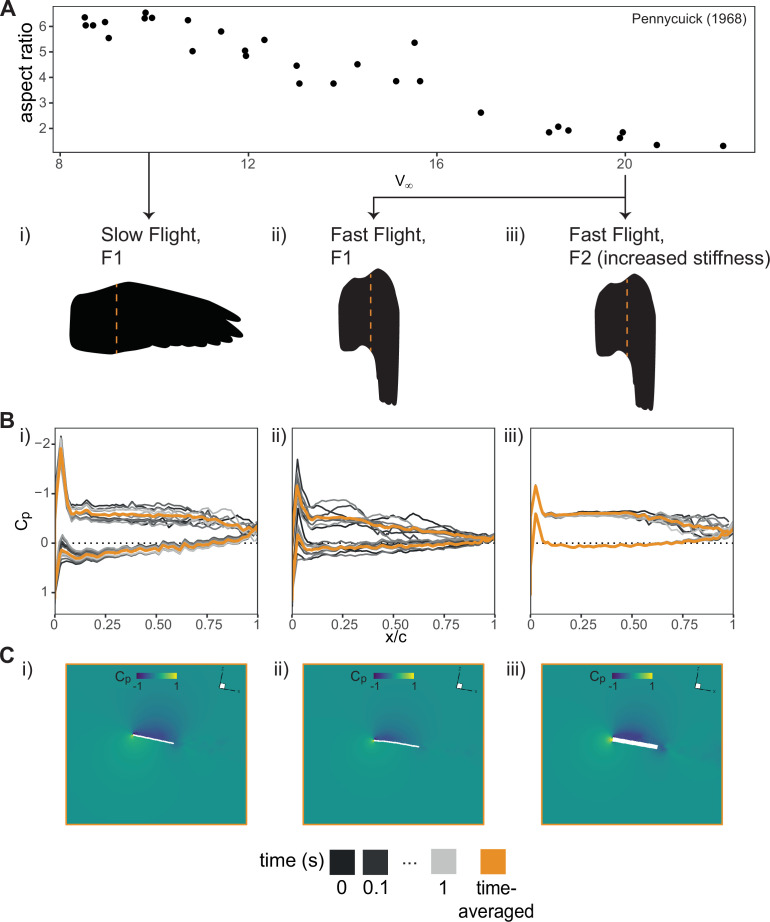
Coupling an increase in stiffness with wing folding during high-speed flight prevents destructive aeroelastic flutter that may reduce time-averaged total lift. (A) Three simulated cases with wing shapes corresponding to slow and fast flight (adapted from [2]) and their prescribed stiffness: (i) the extended wing with low stiffness $\left(F1,E=10^{6}\right)$, (ii) the folded wing with low stiffness $\left(F1,E=10^{6}\right)$ and (iii) the folded wing with moderate stiffness $\left(F2,E=10^{9}\right)$. (B) Chordwise time-dependent pressure (*Cp*) distributions (black to grey) and time-averaged *Cp* (orange) show a reduced low-pressure region over the trailing edge at certain time points. (C) This is reflected in the time-averaged *Cp* contour plots.

We propose that the stability of near-wall vortex structures is sensitive to differences in the timing and amplitude of the dynamic aeroelastic responses caused by the combination of wing shape, flexibility and speed. At 10 m s^−1^, the extended F1 wing used for flying at these speeds [2] oscillated at low frequencies but high amplitudes (electronic supplementary material, figure S5). This deformation was synchronous with the small vortex shed from the leading edge and it could be observed to roll along the dorsal surface towards the trailing edge within one cycle (figure 4A(i) and electronic supplementary material, video S6a). At 20 m s^−1^, the speed at which birds tend towards folded wing configurations [2], the flexible F1 folded wing continued to oscillate at high amplitudes but also at higher frequencies (electronic supplementary material, figure S5). Furthermore, the chordwise mode shape of this oscillation also differed compared with the extended F1 wing at slow speeds. The wing then deformed in a way that seemed to destructively interfere with the leading-edge vortex and we could no longer observe a low-pressure region towards the trailing edge of the wing (figure 4A(ii) and electronic supplementary material, video S6b). A reduction of the pressure gradient over the entirety of the wing area means that there was less aerodynamic force, and therefore less overall generated lift. By increasing the stiffness, the F2 folded wing deformed at a much lower amplitude (electronic supplementary material, figure S5). This change in the aeroelastic deformation in these stiffer wings is reflected in other studies on flow-induced vibrations of flexible plates [47]. As a result, the detrimental effect of the large deformations experienced by the F1 folded wing was no longer present and a pressure gradient could be observed in the entirety of the wing’s chord (figure 4A(iii) and electronic supplementary material, video S6c).

To summarize, we determined that a feathered wing can experience large increases in stiffness due to feather–feather interaction when folding for high-speed flight. This coupled change in the mechanical properties of the wing may provide a mechanism to further increase the aerodynamic performance envelope by tuning the aeroelastic response to alter local flow and vorticity.

## 4. Discussion

Wing morphing provides birds with the ability to increase their performance envelope, adapting to changing functional needs and environmental conditions during flight. While the effects of wing shape on aerodynamic performance [2–7] have been previously reported, there has been limited investigation on how the rearrangement and interaction of the wing structures, each with their own material and structural properties [13–15,48], during morphing can result in aeroelastic-based effects on local flow and modulation of local aerodynamic forces. In this study, we use experimental and computational techniques to explore, in particular, the contributions of feather rearrangement during wing folding on these aeroelastic-based effects. From our results, we suggest that the coupling of overlapping feathers to the movement of skeletal elements [12] during wing folding can locally increase stiffness (figure 2), among other mechanical effects, which may alleviate a loss of lift (figure 3) due to detrimental aeroelastic deformations (figure 4) during high-speed gliding flight.

Fluid–structure interaction simulation has allowed us to test combinations of wing shape and wing stiffness that either have or have not been observed in natural birds. This allows us to relate our results with *in vivo* experimental studies as well as explore the parameter space beyond what is available in nature. In all cases, our $C_{L}$ and $C_{D}$ data fall within the expected range of *in vivo* pigeon flight, but the mean aerodynamic coefficients of our flexible model were closer to those reported by Pennycuick [2] compared with the rigid model. This suggests that despite past focus on the effects of propulsor flexibility on flight performance during flapping flight [29,49–51], accounting for the aeroelastic deformation of the wing even in fixed-wing gliding flight can have noticeable effects on the predicted aerodynamic forces. In particular, the magnitude of the effects of wing flexibility varies based on the overall wing shape and can be more significant for a given shape than the more commonly considered parameter of flight speed (figure 3).

Visualization of the simulation results reveals that the increase in lift and decrease in drag of the flexible wings are due to a reorientation of the pressure gradient in a more dorsal-ventral direction (figure 3C and electronic supplementary material, figure S53. As the flexible wing deforms in both plunge and twist motions (electronic supplementary material, figure S5), it interacts with the separating boundary layer. This oscillation increases the vorticity (figures 3D and electronic supplementary material, figure S4) and energy mixing into the near-wall flow, thus preventing flow separation near the wing surface. Lift enhancement due to flow-induced vibrations or prescribed oscillatory motions on airfoil modifying the pressure distribution has been reported for low Reynolds numbers in both numerical [52,53] and experimental studies [54]. Despite operating at the moderately larger Reynolds numbers experienced by gliding birds, their flexible wings may provide a similar improvement through vorticity-based effects.

Furthermore, we show that this lift enhancement is dependent on both wing shape and flexibility (figure 3A) due to the synchronization of the aeroelastic oscillations with the frequency of vortices shed from the leading edge. Performance enhancement through the synchronization of oscillatory behaviour with vortex shedding has been previously studied in the context where vortices are generated by an upstream obstacle. In those cases, tuning the oscillatory behaviour through animal behaviour [55] or through structure flexibility [56,57] is used to minimize vortex-induced force fluctuations. We find that synchronizing the oscillatory aeroelastic responses of the wing in phase with self-generated vortices can also result in these leading-edge vortices moving downstream to maintain the low-pressure area over the surface (figure 4 and electronic supplementary material, video S6c), a phenomenon referred to as ‘frequency lock-in’ [52,53].

Our simulations show that certain combinations of wing shape, flexibility and airspeed show this beneficial phenomenon but others do not. The highly flexible folded wing used for high-speed gliding flight deforms out of sync and destructively interferes with the leading-edge vortices and prevents a low-pressure area from forming over the trailing edge of the wing (figure 4 and electronic supplementary material, video S6b). Our study proposes that this detrimental effect can be alleviated by coupling feather movement with wing morphing to stiffen the proximal region of the wing (figure 2B), where much of the lift is generated in gliding flight [58,59]. Contrary to bats, which modulate wing stiffness actively using muscles embedded throughout the wing membrane [60], the change in wing stiffness measured in this study is entirely due to the anatomical arrangement of the wing: distal feathers near the leading edge are attached more firmly to the digits 2 and 3 via phalangoremigial ligaments compared with proximal feathers that have attachments to other soft tissues and neighbouring feather follicles [10]. Aeroelastic control to enhance performance for a flight speed is, therefore, not controlled directly by the bird, but is outsourced to the wing anatomy. This ‘computational morphology’ has the advantage of reducing control complexity [61].

This study offers only a snapshot of the possible mechanisms for aeroelastic control by coupling multiple passive structures to a few active components. While this aeroelasticity-based lift enhancement is unlikely to be essential for steady gliding flight, we predict that experimentally altering the stiffness of the feathered wing independent of the wing shape during *in vivo* gliding flight would result in a loss of aerodynamic efficiency. This might manifest as an increase in metabolic cost as measured by $CO_{2}$ production or as behavioural compensation to the gliding kinematics to overcome the loss in lift. Further work, such as cases with other stiffness values, would allow us to better resolve the finer details of this complex, nonlinear relationship between wing morphing, mechanical properties and fluid–structure effects on the flight. Additionally, a more realistic, multi-material, anisotropic wing model incorporating both local changes in feather–feather interaction as well as activation of small feather muscles [10] may provide insight on how wing structures can contribute to tuning aeroelastic response for flow control during flight. To say nothing of the work needed to understand the effect of other morphing modalities, dynamic aerodynamic forces and interspecific differences.

In conclusion, the anatomical arrangement of the avian feathered wing provides a mechanism in which coupled movements of passive elements indirectly modulate local aeroelastic effects for flow control with minimal computational complexity. This is a foundational study that aims to fill a gap in knowledge on the mechanical and aeroelastic contributions of wing morphing to aerodynamic performance in birds. By integrating biological experiments and fluid–structure interaction modelling, we show that wing folding for high-speed gliding flight increases stiffness in the proximal wing through feather–feather interaction, and this may restrict lift-reducing aeroelastic flutter modes at those speeds. Understanding how biomechanical mechanisms affect flight control is critical for making informed bioinspired design decisions for small uncrewed aerial vehicles (UAVs), and one area that has been sparsely studied is the role of aeroelasticity [62]. Our study shows that controlling boundary layer vorticity, an important feature in aircraft technology [63], can be achieved without the weight of additional actuators, sensors or computational complexity. This is a highly beneficial advantage due to the small size of many UAVs. Lastly, while this study shows one case of how feather rearrangement can tune aeroelastic response for improved performance at different speeds, this concept could be expanded for other functions such as flow sensing [64] and broadband energy harvesting [65] applications as well.

## Data Availability

The data and scripts that support the findings of this study are openly available in Figshare [66]. Supplementary material is available online [67].
